# Fluorescent Biosensor Imaging of Nitrate in *Arabidopsis thaliana*

**DOI:** 10.21769/BioProtoc.4743

**Published:** 2023-08-20

**Authors:** Yen-Ning Chen, Cheng-Hsun Ho

**Affiliations:** Agricultural Biotechnology Research Center, Academia Sinica, Taipei, Taiwan

**Keywords:** Nitrate, Genetically encoded biosensor, Yeast transformation, *Arabidopsis thaliana*, Light-sheet imaging system, Visualization

## Abstract

Nitrate (NO_3_^–^) is an essential element and nutrient for plants and animals. Despite extensive studies on the regulation of nitrate uptake and downstream responses in various cells, our knowledge of the distribution of nitrogen forms in different root cell types and their cellular compartments is still limited. Previous physiological models have relied on in vitro biochemistry and metabolite level analysis, which limits the ability to differentiate between cell types and compartments. Here, to address this, we report a nuclear-localized, genetically encoded fluorescent biosensor, which we named nlsNitraMeter3.0, for the quantitative visualization of nitrate concentration and distribution at the cellular level in *Arabidopsis thaliana*. This biosensor was specifically designed for nitrate measurements, not nitrite. Through genetic engineering to create and select sensors using yeast, *Xenopus* oocyte, and *Arabidopsis* expression systems, we developed a reversible and highly specific nitrate sensor. This method, combined with fluorescence imaging systems such as confocal microscopy, allows for the understanding and monitoring of nitrate transporter activity in plant root cells in a minimally invasive manner. Furthermore, this approach enables the functional analysis of nitrate transporters and the measurement of nitrate distribution in plants, providing a valuable tool for plant biology research. In summary, we provide a protocol for sensor development and a biosensor that can be used to monitor nitrate levels in plants.

Key features

This protocol builds upon the concept of FRET biosensors for in vivo visualization of spatiotemporal nitrate levels at a cellular resolution.

Nitrate levels can be quantified utilizing the biosensor in conjunction with either a plate reader or a fluorescence microscope.

Graphical overview


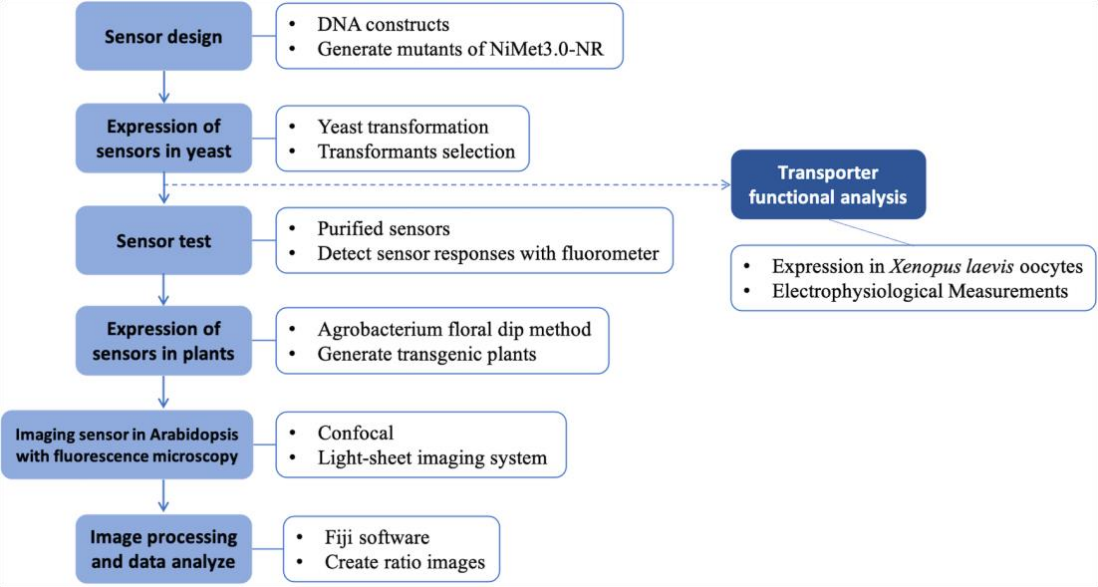


## Background

Genetically encoded sensors have been developed over the past decade. The first fluorescence protein–based calcium sensor, Cameleon, was created and utilized to monitor calcium signaling processes in plant stomata (Allen et al., 2001). Since then, other sensors have been developed and applied in both plant and animal biology. One example is the glucose sensors FLIPglu600μΔ13V and FLIPsuc90μ∆1V, which were created by Chen et al. (2010 and 2012, respectively). These optical sensors have proven useful not only for monitoring analyte levels and fluxes but also for identifying elusive sucrose efflux transporters required for phloem loading. Overall, genetically encoded sensors have proven to be highly versatile tools with broad applicability in a range of research settings.

In our study, we developed a Förster resonance energy transfer (FRET)-based nitrate sensor, named nlsNitraMeter3.0, to successfully monitor the steady-state levels, accumulation, and dynamic conditions of nitrate distribution and content in *Arabidopsis thaliana* through fluorescence confocal microscopy. Using this sensor, we can directly visualize the spatial and temporal distribution of nitrate with high resolution at the cellular level. In addition, the sensor can be used to acquire images through fluorescence microscopy before and after treatment with different media. Through image analysis software (e.g., Fiji), the images can be quantified. Furthermore, the concept and operation of nlsNitraMeter3.0 can be applied to develop new sensors that identify or characterize other molecules in plants. For instance, we previously reported that NPF1.3 plays a role as a nitrate transporter by some functional in vitro analysis (e.g., *Xenopus laevis* oocytes) (Chen and Ho, 2022).

Here, we provide the principles of engineering, detection methods, and application of nlsNitraMeter3.0. By following the protocol reported herein it will also be possible to develop further sensors (refer to Supplementary information).

## Materials and reagents

**Biological materials**

1. Yeast strain: protease-deficient yeast strain BJ5465 (MATa, *ura3–52, trp1, leu2Δ1, his3Δ200, pep4::HIS3, prb1Δ1.6R, can1*, GAL+) (ATCC, catalog number: 208289TM), which was obtained from the Yeast Genetic Stock Center (University of California, Berkeley, CA).
2. Sensors: NIT domain/NasR
  The full-length open reading frame of NasR from *Klebsiella oxytoca* (Boudes et al., 2012) in the pDONR221 GATEWAY Entry vector was used as a sensory domain to create the nitrate sensors NiMet3.0 and nlsNiMet3.0. *Note: Constructs were inserted using an Entry clone by Gateway LR reactions into the yeast expression vectors pDRFlip30.*
  The NiMet3.0 is fused to the full-length of NasR with pDRFlip30 vector and sandwiched between an N-terminal Aphrodite t9 (AFPt9) variant (Deuschle et al., 2006), with nine amino acids truncated off the C terminus, and a C-terminal monomeric Cerulean (mCer) (Rizzo et al., 2006).
*Note: The pDRFlip30 vector was modified from pDRFlip39 vector (Addgene, catalog number: 65517).*
3. Agrobacterium strain: *Agrobacterium tumefaciens* strain GV3101 was used here to obtain high transformation rates and high levels of expression, typically leading to high copy insertion into the genome (Koncz and Schell, 1986).
4. *Arabidopsis thaliana:* wild type Col-0, a nitrate transporter mutant [npf6.3/NTR1;1/chl1-5 (Leran et al., 2014)], and a nitrate reductase mutant [nia1nia2 (Desikan et al., 2002)] were used.

**Reagents**

1. Potassium nitrate (KNO_3_) (Sigma-Aldrich, catalog number: P8394)
2. Potassium chlorate (KClO_3_) (STREM, catalog number: 93-1913)
3. Potassium chloride (KCl) (Sigma-Aldrich, catalog number: P5405)
4. Potassium nitrite (KNO_2_) (ARO, catalog number: 42306)
5. Potassium sulfate (K_2_SO_4_) (SHOWA, catalog number: 1648-4150-000-23)
6. Potassium sulfite (K_2_SO_3_) (ACROS, catalog number: 44021)
7. Potassium selenite (K_2_SeO_3_) (STREM, catalog number: 931971-000000-18)
8. Potassium molybdenum oxide, anhydrous (K_2_MoO_4)_ (Alfa, catalog number: 22898-0000000-17)
9. Ammonium chloride (NH_4_Cl) (Merck, catalog number: 1.01145-0500)
10. Magnesium chloride hexahydrate (MgCl_2_.6H_2_O) (Sigma-Aldrich, catalog number: M9272)
11. Gly-Gly (Sigma-Aldrich, catalog number: G1002)
12. YNB, yeast nitrogen base w/o amino acids w/o ammonium sulfate (BD, Difco, catalog number: 233520)
13. DO supplement-Ura (Takara Bio Company, Clontech, catalog number: 630416)
14. D-(+)-Glucose monohydrate (Fluka Analytical, catalog number: 49159)
15. Sucrose (Merck, catalog number: 1.07687.1000)
16. Agar (BD Bacto^TM^, catalog number: DIF214530)
17. Agar (Phytoagar^TM^, catalog number: 40100072)
18. MES hydrate (Sigma-Aldrich, catalog number: M2933)
19. MOPS (Sigma-Aldrich, catalog number: M3183)
20. Sodium hydroxide (NaOH) (Sigma-Aldrich, catalog number: S5881)
21. 1, 4-Dithiothreitol (DTT) (Sigma-Aldrich, catalog number: DTT-RO)
22. Carrier DNA [UltraPure^TM^ salmon sperm DNA solution (Thermo Fisher Scientific, Invitrogen^TM^, catalog number: 115632-011)]
23. Ethylenediaminetetraacetic acid (EDTA) (Sigma-Aldrich, catalog number: E6758)
24. Polyethylene glycol 4000 (PEG4000) (Fluka Analytical, catalog number: 81240)
25. Lithium acetate dihydrate (LiOAc) (Sigma-Aldrich, catalog number: L4158)
26. Tris hydrochloride ultrapure bioreagent (Tris-Cl) (J.T. Baker, catalog number: 4103-02)
27. Sodium chloride (NaCl) (Merck, catalog number: 1.06404.1000)
28. Sodium hydroxide (NaOH) (Merck, catalog number: 1.06498.1000)
29. Peptone (BD Bacto^TM^, catalog number: 211677)
30. Yeast extract (BD Bacto^TM^, catalog number: 212750)
31. MS modified basal salt mixture without nitrogen (MS) (PhytoTech Labs, catalog number: M531)
32. MilliQ or distilled water
33. Spectinomycin
34. Kanamycin

**Solutions**

1. 40% (w/v) glucose solution (sterile and filtrated)
2. MOPS buffer
3. MES buffer
4. Yeast extract peptone dextrose (YPD) medium (see Recipe 1)
5. Solid yeast nitrogen base (see Recipe 2)
6. PLATE mixture (the acronym of PEG, lithium acetate, Tris, and EDTA) (see Recipe 3)
7. Wash buffer (see Recipe 4)
8. Resuspension buffer (see Recipe 5)
9. Substrate addition (see Recipe 6)
10. Plant growth solid base (see Recipe 7)

**Recipes**

1. **Yeast extract peptone dextrose (YPD) medium** ^a, c^
  **Table:** 

Reagent	Final concentration	Amount
Peptone	2.0% (w/v)	10 g
Yeast extract	1.0% (w/v)	5 g
Agar	2.4% (w/v)	12 g
40% sterile filtrated glucose^b^	2% (w/v)	25 mL
H_2_O	n/a	n/a
Total	n/a	500 mL
  - Autoclave, 121 °C, 15 psi, 15 min
  - For liquid medium, when hand-warm, add glucose from 40% sterile filtrated stock to a final concentration of 2% under a sterile hood (e.g., biosafety cabinet).
  - For solid medium, add 20 g/L agar before autoclaving. Add sterile filtrated glucose from 40% stock to a final concentration of 2% when the medium is hand-warm before pouring plates.
2. **Solid yeast nitrogen base (-*ura* DropOut medium)** ^a, c^
  **Table:** 

Reagent	Final concentration	Amount
Yeast nitrogen base w/o amino acids w/o ammonium sulfate	1.7 g/L	1.7 g
DO supplement-Ura ^d^	0.77 g/L	0.77 g
40% sterile filtrated glucose^b^	2% (w/v)	50 mL
H_2_O	n/a	to 1,000 mL
Total	n/a	1,000 mL
  - Autoclave, 121 °C, 15 psi, 15 min
  - For liquid medium, when hand-warm, add glucose from 40% sterile filtrated stock to a final concentration of 2% under a sterile hood (e.g., biosafety cabinet).
  - For solid medium, add 20 g/L agar before autoclaving. Add sterile filtrated glucose from 40% stock to a final concentration of 2% when the medium is hand-warm before pouring plates.
  - Adjust the pH of the *-ura* DropOut medium to pH 5.8 with NaOH before addition of agar and autoclaving.
3. **PLATE mixture for yeast transformation**
  **Table:** 

Reagent	Amount
45% PEG4000	90 mL
1 M LiOAc	10 mL
1M Tris-Cl (pH7.5)	1 mL
0.5M EDTA	0.2 mL
Total	100 mL
4. **Wash buffer**
  **Table:** 

Reagent	Final concentration	Amount
MES	50 mM	9.76 g
Total	n/a	1,000 mL
  - Adjust the pH of the MES buffer to pH 5.5 with NaOH and autoclave.
  - Autoclave, 121 °C, 15 psi, 15 min.
5. **Resuspension buffer**
  **Table:** 

Reagent	Final concentration	Amount
MES buffer (Recipe 4)	50 mM	250 mL
Agar	0.05%	0.125 g
Total	n/a	250 mL
  - Wait until the medium cools to room temperature (RT) to delay sedimentation of the cells during the measurement.
6. **Plant growth solid base^a–d^**
  **Table:** 

Reagent	Final concentration	Amount
MS salts without nitrogen	1/2 strength	0.78 g
20% sterile filtrated sucrose	0.5 % (w/v)	25 mL
Total	n/a	1,000 mL
  - Adjust the pH to pH 5.5 with KOH before addition of agar and autoclaving.
  - Autoclave, 121 °C, 15 psi, 15 min
  - For liquid medium, when hand-warm, add sucrose from 20% sterile filtrated stock to a final concentration of 0.5% under a sterile hood (e.g., biosafety cabinet).
  - For solid medium, add 12 g/L Phytoagar^TM^ before autoclaving. Add sterile filtrated glucose from 20% stock to a final concentration of 0.5% when the medium is hand-warm before pouring plates.
7. **Substrate addition**
  **Table:** 

Reagent	Final concentration	Amount
KNO_3_ or other substrates (Reagent 1–11)	Depending on the experimental design	-
MES buffer	50 mM	-
Total	n/a	50 mL
  Depending on the concentration of the nitrate needed, use the nitrate stock solution and dilute it with MES buffer or MOPS buffer.

**Laboratory supplies**

1. 96-well microplates (flat bottom clear or black) (Greiner Bio-One, catalog numbers: 650101 and 650209)
*Note: Black plates have a lower background.*
2. Multichannel (12) pipette (for 100 μL) (e.g., Sartorius, catalog number: 725240)
3. 50 mL sterile plastic tubes (Falcon^®^)
4. Petri dishes (diameter: 9 mm; height: 15 mm; sterile) (Alpha Plus, catalog number: BL6905)
5. Glass beads (3 mm) (BasicLife, catalog number: CEO-1169)
6. Vacuum-driven filter system (250 mL, upper cup, 0.22 μm PES) (AGC, catalog number: AGC-VC-PES22-250-1CS)

## Equipment

1. Monochromator-based spectrofluorimeter for 96-well plates [Safire or Infinite^®^ M1000 (Tecan Trading)]
2. Cell density meter (Amersham, model: Ultrospec 10)
3. Orbital shaker, with temperature and velocity control (Eppendorf, New Brunswick Scientific, model: Innova 44)
4. Incubator for 28–30 °C incubation of yeast cells (YIHDER, model: LM-570RD)
5. Centrifuge with swinging rotor for 50 mL tubes (Eppendorf, model: 5810R)
6. Inverted confocal plus super resolution microscope (Zeiss, LSM 780 + ELYRA): high sensitivity confocal microscope is equipped with GaAsP spectrum detector, and the super resolution microscope is a Structure Illumination Microscopy.
7. The laboratory-established light-sheet system was made in cooperation with Microlambda Pte Ltd (Singapore)
8. Growth chamber

## Software and datasets

1. MetaMorph software (Downingtown, PA)
2. Fiji (http://fiji.sc/)
3. GraphPad Prism version 9.0.0 for Mac (www.graphpad.com)

## Procedure

- **FRET sensor design**
  For a detailed account of the generation of the sensor DNA constructs and the sensor mutants, please refer to Chen and Ho (2022). In this section, we just report a few concepts of the sensor design.
  1. DNA constructs
    Based on the FRET characteristic, we designed the Gateway expression clones with an insert of the bacterial (*K. oxytoca*) NasR/NIT domain (Figure 1).
    **Figure 1.**
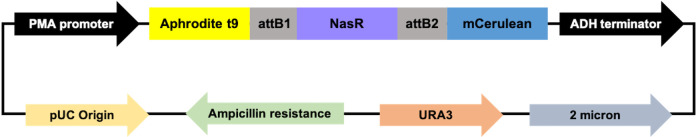
Map of pDRFlip30-NasR plasmids. NasR, an NO3-binding protein, was fused via attB1 and attB2 linkers to a fluorescent protein FRET pair (donor: Aphrodite, and acceptor: Cerulean). The NasR protein (purple) representation is from a published structure of NasR [PDB 4AKK (Boudes et al., 2012)]. The Aphrodite (yellow) representation is from a published structure of Venus [PDB 1MYW (Rekas et al., 2002)] and the Cerulean (blue) representation is from a published structure of Cerulean [PDB 2WSO (Lelimousin et al., 2009)].
- **Expression of sensors in yeast and fluorescence analysis**
  1. Yeast transformation
    The protease-deficient yeast strain BJ5465 is transformed with the sensors containing the desired above (e.g., NiMet-NIT, NiMet1.0, NiMet2.0, NiMet3.0, nlsNiMet3.0, or NiMet3.0-NRs) by using the modified lithium acetate method from Gietz et al. (1992). In brief:
    - Inoculate cultures in YPD medium and grow at 30 °C overnight to absorbance ~0.5 at OD 600 nm.
    - Spin down (2,000× *g*) 1 mL of cells in a microfuge tube (15 s) for each transformation.
    - Decant the supernatant and resuspend the cells in 100 μL of YPD medium by vortexing.
    - Add 2 μL of 10 mg/mL carrier DNA and vortex.
    - Add ~1 μg plasmid and vortex.
    - Add 0.5 mL of *PLATE mixture* [100 mL stock containing 90 mL of 45% PEG4000, 10 mL of 1 M lithium acetate, 1 mL of 1 M Tris-Cl (pH 7.5), 0.2 mL of 0.5 M EDTA] and vortex.
    - Add 20 μL 1 M DTT and vortex.
    - Incubate at 25 for 6–8 h or overnight.
    - Heat-shock cells for 10 min at 42 °C.
    - Place a pipette tip directly into the bottom of the tube, withdraw 50–100 μL of cells, and plate cells on solid -*ura* DropOut medium. Wrap plates with plastic cling wrap to prevent dehydration.
    - Incubate plates (lid down) at 30 °C for 2–3 days.
  2. Detection of NitraMeter responses in yeast using a fluorimeter
    - Select transformed yeast on solid YNB supplemented with 2% glucose and -*ura* DropOut medium.
    - Pick single colonies by using sterile pipette tips and grow in a 50 mL tube containing 10 mL of -*ura* DropOut liquid medium. Pick at least three independent colonies.
*Note: Use fresh transformation. To avoid mutations in yeast or plasmid, do not keep colonies for more than one week on plates.*
    - Place tubes in a rack and incubate in an incubator for ~15 h under agitation (220 rpm) at 30 °C until the culture reaches absorbance ~0.5 at OD_600nm_.
    - Subculture liquid cultures after dilution to OD_600nm_ 0.01 in the same liquid medium and grow at 30 °C until absorbance reaches ~0.3 at OD_600nm_.
    - Collect the cells by centrifugation at 3,000× *g* for 10 min at RT to precipitate the cells.
    - Discard the supernatant and resuspend the precipitate by vortexing in 10 mL of wash buffer for 15 s at RT.
    - Centrifuge at 3,000× *g* for 10 min at RT again.
    - Wash the precipitate two more times as in Step e–f to remove traces of growth medium.
    - Resuspend the precipitate to absorbance ~0.5 at OD_600nm_ in resuspension buffer.
    - Mix cells well and aliquot 100 μL of the culture into wells of a 96-well flat bottom plate.
    - Measure the fluorescence in a fluorescence plate reader in bottom reading mode using 7.5 nm bandwidth for both excitation and emission. Typically, emission spectra are recorded with the following instrument settings: λem 470–570 nm for donor (mCer), step size 5 nm, gain: 75; and λem 520–570 nm for AFPt9, step size 5 nm, gain: 75. Fluorescence from pDRFlip30 (donor, mCer), pDRFlip39 (donor, t7.ed.eCFPt9), and pDRFlip42-linker (donor, mCer) was measured by excitation at λexc 428 nm; AFPt9 is measured with excitation at λexc 500 nm.
    - Use a single or multichannel pipette to add 100 μL of the culture to wells (mix by pipetting up and down) and to add analyte solution to the cells. Set up at least three replicates per treatment. Try to add equal volumes of solutions to reduce variability and use well-calibrated pipettes, since the assays are quantitative and sensitive to differences in volumes/concentration of sensor and analyte.
    - Record the fluorescence immediately (as fast as possible) after addition of substrate or control solution. It takes approximately 10 min to read a full 96-well plate with the parameters mentioned above. For highly accurate analyses, measure only a few wells at a time to reduce differences in analysis time. It is also possible to use instruments with injectors that allow for immediate recording; use rapid switching between wells to record over time.
*Note: The sensor exhibits functional activity when employed as a purified recombinant protein.*
- **Expression of NiMet3.0, NiMet3.0-NR-R176A, and nlsNiMet3.0 in *Arabidopsis***
  1. DNA constructs for expressing sensors in plants
    - Insert open reading sequence of NasR or NasR-NR-R176A into the multiple cloning site of the p16-Kan vector (Jones et al., 2014): 5′-, a sequence coding for the SV40-derived nuclear localization signal LQPKKKRKVGG (Schuster et al., 2014); a sequence coding for Aphrodite; a Gateway cassette including attR1, Chloramphenicol resistance gene, ccdB terminator gene, and attR2; a sequence coding for mCerulean (mCer); and a sequence coding for the cMyc epitope tag -3′, or pZPFlip UBQ10-KAN vector under the control of the UBQ10 promoter.
*Note: The p16 promoter (Schuster et al., 2014) from the AT3G60245 gene encoding a 16S ribosomal subunit was used to drive the nuclear-localized NiMet3.0 fusion biosensor, whereas the CaMV 35S promoter (Battraw and Hall, 1990) was used to drive the NiMet3.0 and NiMet3.0-NR-R176A fusion biosensor in plants.*
    - Recombine in Gateway LR reactions with NasR or NasR-NR-R176A Entry Clones, resulting in NiMet3.0, NiMet3.0-NR-R176A, and nlsNiMet3.0 expression clones.
  2. Generate transgenic plants using the *Agrobacterium* floral dip method
    - Introduce sensors into *Agrobacterium tumefaciens* GV3101.
    - Grow healthy *Arabidopsis* plants in 12 h of light, 50% humidity, and at 22 °C until they begin to bolt and produce floral inflorescences (3–4 weeks in a growth chamber).
    - Remove siliques and mature flower clusters before floral dipping.
    - Inoculate a single *Agrobacterium* colony that was transformed with sensors into 5 mL of liquid LB medium containing the appropriate antibiotics [spectinomycin (final concentration 100 μg/mL)] for binary vector selection. Incubate the culture at 28 °C overnight.
    - The following morning, use this feeder culture to inoculate 200 mL of liquid LB with spectinomycin (final concentration 100 μg/mL) and grow the culture at 28 °C for 16–24 h.
    - Collect *Agrobacterium* cells by centrifugation at 3,000× *g* for 10 min at RT and discard the supernatant. Then, gently resuspend cells in one volume of the freshly made dipping medium.
    - Dilute *Agrobacterium* cells to 6 × 10^9^ cells/mL.
    - Spray the *Agrobacterium* on the floral part of the *Arabidopsis*. Then, lay down the dipped plants in a plastic basin and cover them with plastic wrap for 16–24 h to maintain high humidity.
    - The next day, remove the cover and allow them to grow normally for one month in the greenhouse or the growth chamber; withhold watering when siliques turn brown.
  3. Select transformants on agar plates containing 1/2× MS medium with vitamins (PhytoTech Labs, M519) and kanamycin (30 mg/L).
- **Imaging the nitrate sensor in *Arabidopsis* with fluorescence microscopy confocal microscopy**
*Note: Although a fluorescence confocal microscope is the standard equipment used, light-sheet microscopy is another option. The settings for laser intensity, detector, and objective are similar to those for confocal microscopy. Please refer to the detailed procedure of the light-sheet system in the Supplementary information section.*
  1. Germinate and grow transgenic seedlings
    - Germinate and grow vertically on 1/2× MS modified basal salt mixture without nitrogen, 1% agar, and 0.05% (w/v) sucrose (pH 5.7) plates.
    - Place 5- or 6-day-old seedlings in the solution containing 1/2× MS medium [1/2× MS and 0.05% sucrose (pH 5.7)] and prepare for imaging on glass slides.
  2. Nitrate treatments on glass slides for confocal microscopy
    - Place seedlings on glass slides with 50 μL of solution, surround with a rectangle of vacuum grease, and cover with a square coverslip equal in height and half the width of the vacuum grease rectangle.
    - Exchange the nitrate treatment solution beneath the coverslip by addition to the left and removal from the right side of the coverslip.
    - Acquire confocal images on a Zeiss 780 laser scanning microscope and use a 20×/0.8 Plan-Apochromat dry objective or 40×/1.2 C-Apochromat water objective. Excite CFP (440 nm) and yellow fluorescent protein (YFP; 514 nm) with lasers. Detect fluorescence emission using a GaAsP photomultiplier tube (PMT) detector, set to detect 463–508 nm for CFP, and a normal PMT detector, set to 520–585 nm for YFP. Set the laser power between 0.5% and 2% with detector gain set to 700–750 to image CFP or YFP.
    - Acquire images at time points based on the purpose of the research (refer to the note below for details on time point settings). Acquire three-dimensional images, with a z-step size of 1.5 μm and half the diameter of the primary root axis in *Arabidopsis*.
*Notes:*
*i. For example, if the purpose is to observe the nitrate distribution in the root after different concentrations of medium supplement, it is suitable to set the range and interval of the time points to less than an hour, unless the sample can be kept moist. Additionally, fluorescence blenching should be considered when continuously recording.*
*ii. For other methods that can be used to obtain continuous images or video, please refer to the Supplementary information.*

## Data analysis

- **Fluorescence emission ratio response of purified NiMet3.0 to NO_3_**^−^ **in vitro**
  1. Subtract background fluorescence of yeast (using cells transformed with vector only) from all fluorescence values (for both spectra as well as single point measurements).
  2. The solution addition might trigger a change in the energy transfer rate between the emission at 530 nm [Dx acceptor emission (DxAm)] and the emission at 488 nm [Dx donor emission (DxDm)] that could act as a FRET ratio change sensor (ΔDxAm/DxDm). Through several optimizations, we obtained a fusion construct that shows a significantly substrate-triggered positive ratio change (ΔDxAm/DxDm) (e.g., NiMet3.0) (Figure 2).
*Notes:*
    - *NiMet3.0 expressed in yeast responds to nitrate addition by changing the fluorescence intensity of donor and acceptor emission (obtained with excitation at 428 nm). Aphrodite-t9 emission was unaffected and served as a control or reference for normalization (obtained at 500 nm excitation). Nitrate addition (5 mM) induced a decrease in the emission spectrum of the donor, and the emission of the acceptor increased (Figure 2A). Besides, since the Aphrodite-t9 emission is unaffected by nitrate when excited directly, Aphrodite-t9 emission can be used as a control and for normalization by using ratios instead of absolute values to compare between different cultures.*
    - *The various nitrate concentrations from micromolar to millimolar were added externally to the primary root to monitor the NitraMeter sensor responses. The data showed that the FRET ratio changed to external nitrate addition was saturated after approximately 0.25–0.5 mM, indicating either that the NitraMeter sensor in root was all occupied by nitrate or the Vmax of NitraMeter sensor was reached after the concentrations of nitrate addition externally.*
    **Figure 2.**
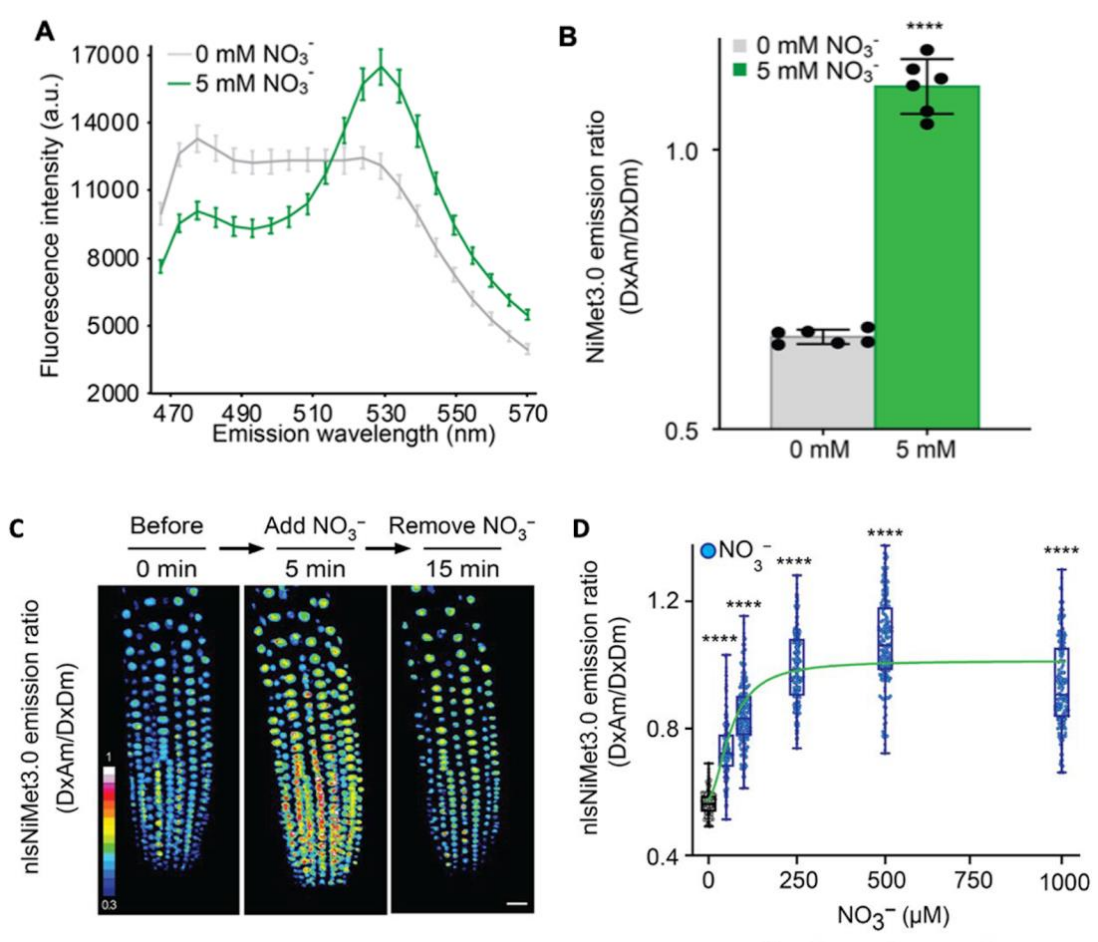
Fluorescence response of NiMet 3.0- and nlsNiMet 3.0-expressing yeast cells and *Arabidopsis* root. (A) Fluorescence emission wavelength scan (B) and emission ratio at 530 nm of purified NiMet3.0 protein with and without NO_3_^−^. Nitrate concentration is indicated in the figures. Nitrate was able to trigger responses that were significantly different from the control (*, P < 0.0001, *t*-test). The presented data are mean ± SD of six biological repeats. (C) Three-dimensional images of nlsNiMet3.0 emission ratios of 5-day-old root meristem zone in transgenic Col-0 before a NO_3_^−^ pulse, after the NO_3_^−^ pulse, and after removing the NO_3_^−^. NO_3_^−^ (50 μM) was used. (D) Beeswarm and box plot of NO_3_^−^ concentration-dependent nlsNiMet3.0 emission ratios for nuclei of root tips. ****, P < 0.0001, Student’s *t*-test. Means ± SD of three biological repeats are presented.
- **Quantification of the ratio of the fluorescence pixel intensity of the nitrate sensor in vivo**
  1. Use Fiji software to process the image and quantify the fluorescence pixel intensity. Calculate the mean gray values of regions of interest (ROIs) within the root meristem region.
    - Subtract the background from all measured intensities generated by ROIs where there was no plant material.
    - Measure the mean intensity values in all four channels (Dx/Dm, Dx/Am, Ax/Dm, and Ax/Am), and subtract that intensity from the entire image.
    - Create ratio images (DxAm/DxDm) by using the Ratio Plus plug-in for ImageJ (P. Magalhães, University of Padua, Italy) (Figure 2C). Select and analyze ROIs with the help of the ROI manager tool.
  2. Import the fluorescence pixel intensity to GraphPad Prism to generate figures (e.g., Figure 2D) and present the data following the same setting rules as described below (see General notes).

## General notes and troubleshooting

1. Despite the NitraMeter3.0 being able to visualize the nitrate dynamic within the cells in *Arabidopsis thaliana*, investigation of nutrient acquisition has relied heavily on techniques that integrate uptake over the entire root system. It is worth to note that the responses of NitraMeter to nitrate indicate the net fluxes of nitrate within where the NitraMeter is located in cell. In addition, net fluxes of NO_3_– into the roots vary both with position along the root axis and with time. These variations may not be consistent in different plants, in which different cells in different roots may not show exact temporal and spatial patterns of nitrate dynamics.
2. Present the data by using beeswarm and box plots of raw data. In the beeswarm and box plot graphs, the central rectangle spans the first quartile to the third quartile, while the line inside the rectangle shows the median. The whiskers denote 1.5 interquartile ranges from the box, and outlying values are plotted beyond the whiskers. Perform statistical analyses using GraphPad Prism version 9.0.0 for Mac (www.graphpad.com).

## Supplementary information

The following supporting information can be downloaded here:
